# Additions to the knowledge of Nevada carabid beetles (Coleoptera: Carabidae) and a preliminary list of carabids from the Great Basin National Park

**DOI:** 10.3897/BDJ.5.e12250

**Published:** 2017-06-13

**Authors:** Kipling Will, Riva Madan, Han Hsuan Hsu

**Affiliations:** 1 University of California, Berkeley, Berkeley, CA, United States of America

**Keywords:** ground beetles, checklist, Western USA beetle fauna

## Abstract

**Background:**

Additions to the list of Carabidae known for Nevada, USA and carabid beetles found in the Great Basin National Park, NV are reported with notes on ecology and identification resources.

**New information:**

For 79 species of carabids, we present 57 new state records, two state records previously reported in online resources, one confirmation of a previous questionable record for the state, and report 22 records for the Great Basin National Park that includes three new state records.

## Introduction

### Carabids of Nevada

Carabidae is family of beetles that have a cosmopolitan distribution and are often very common in the United States. Surprisingly, the number of published records of carabid species from Nevada is staggeringly low. In the recent catalog of North American carabid beetles (Bousquet 2012) the total number of species known for Nevada placed the state as 49th (242 species), only surpassing Delaware (206 species). For comparison, among the 50 US states, Delaware is ranked 49th for total land area, with only 644,718 ha (2,489 sq mi.), while Nevada is ranked 7th with 28,635,092 ha (110,560.71 sq mi.). Compared to adjacent states (California, Oregon, Arizona, Idaho, and Utah) Nevada had only about a third to three quarters the number of species reported. These discrepancies suggested to us that the Nevada fauna was significantly underreported. To better document Nevada’s biodiversity, our study aimed to expand the checklist of the carabid fauna in Nevada by identifying specimens from several western North American Entomological collections and undertaking collecting trips to Nevada.

### The Ecologial Setting in Nevada

The state of Nevada is composed of five ecoregions (U.S. Geological Survey, Department of the Interior, U.S.A 2006, Nevada Fish & Wildlife Office 2014). Three cover the majority of the state: the Northern, Central, and Mojave Basins and Ranges, while two ecoregions, Sierra Nevada Range and Arizona-Mexico Plateau, comprise a relatively small portion of Nevada. Within these ecoregions Nevada’s seasonally hot, dry climate produces a plethora of different ecosystems that support mainly shrubs and grasses at low and medium elevations and conifer trees at higher elevations; however, pockets of isolated environments allow endemic species of plants to thrive and provide habitat for a variety of carabid beetles.

The largest ecoregion in Nevada is the Central Basin and Range. This ecoregion is composed mainly of north-south oriented mountain ranges alternating with dry shrub and grass covered basins. At mid elevations, the mountains are characterised with scattered forests of woodland and mountain brush. Due to the Sierra Nevada Mountains, Nevada’s western region is under a rain shadow and as a result the dry-tolerant piñon-juniper woodlands are common as are stands of ponderosa, lodgepole and white pine. At high elevations (above 2700 m), seasonal moisture supports the growth of high elevation conifers species and aspen groves, with sagebrush and mountain-mahogany in the understory. The precipitation at this elevation also provides perennial streams and ponds with water.

In the Pleistocene much of central Nevada was under what was one of the largest lakes in North America, Lake Lahontan. All that remains of the lake now are playas and a few remnants lakes. The Central Basin and Range is now high desert plain with the exception of small, often seasonal wetlands, typically alkali flats or saline lakes that are replenished by seasonal rains and runoff. In the west and central part of the region there are characteristic rolling valleys with mountian ranges that have alluvial fan outwashes flowing into the lower basins. Diversity in this region is rather low due to its aridity. With a few exceptions, there are almost no continuous woodlands. The highest elevations in this region, those that extend above treeline, are densely covered with mountain big sagebrush, western serviceberry, snowberry, and low sagebrush, but very few trees.

The easternmost part of the Central Basin and Range ecoregion consists of sagebrush valleys, woodlands, mountains, and saline basins. Much of the soil in this region is shallow due to a combination of heavy summer rains and the limestone and dolomite bedrock. As a result, the sagebrush valleys consist of grasses and brush capable of tolerating shallow soils. The woodland zones consist of mainly piñon and juniper forests. Historically, these forests only occurred at higher elevations due to annual natural fires, but fire suppression has allowed the forests to move further down towards the sagebrush valleys. In the more mountainous areas, for example Great Basin National Park (GBNP), various conifers (white fir, Douglas fir, Engelmann spruce, bristlecone pines) dominate. Although high enough to form an alpine zone, climate conditions do not favour the retention of water as most of it rapidly flows out via springs and streams. The GBNP includes a range of the typical floral zones for the region with sagebrush dominated lower elevations, rising through juniper, piñon pine, ponderosa pine, and grassy mountain meadows. Above treeline the habitat is rocky with few plants. Situated on Wheeler Peak, which rises to nearly 4000 m elevation, is a small alpine glacier, the remnant of a more extensive glacier from the last glacial maximum (LGM). Erosional deposition from the glacier's meltwater is evident in much of the of the most-visited parts of the park.

The Northern Basin and Range, which is shared with Oregon and Idaho, is a cooler, more mesic, and less mountainous than the Central Basin and Range. This ecoregion consists of high lava plateaus to the northeast, high lava plains to the northwest and semi-arid uplands scattered across northern Nevada. The high lava plateau receives more precipitation and experiences colder winters resulting in cool season grasses such as bluebunch wheatgrass and Idaho fescue. The high lava plains, a large sagebrush steppe, are very similar to the high lava plateau in climate and vegetation, but have many ephemeral pools that are home to a diverse fauna and flora.

In the far south of Nevada lies the Mojave Basin and Range, a region extending from California to Arizona and southwestern Utah. The warmer, drier climate and milder winters favours a flora dominated by creosote bush at lower elevations. This desert region is far from uniform and is comprised of three subregions: the Amargosa Desert, creosote-bush dominated basins, and arid footslopes. The Amargosa desert is in the rain shadow of the Spring Mountains and is an internally-drained basin with the greatest temperature extremes. Most of the flora is dry adapted creosote bush and bursage. Further east, creosote bush-dominated basins are scattered between the Mojave Desert mountain ranges, grasslands, blackbrush plains, Joshua trees woodlands, and cactus dominated areas. The Spring Mountains prominently rise above the basin habitats with Mount Charleston reaching over 3600 m elevation. Along the elevational gradient various scrub and bush zones lead to juniper, piñon pine, mountain mahogany, culminating in montane conifers.

The Sierra Nevada ecoregion is a mountainous region that is mainly in California but a small part of this ecoregion extends into Nevada along its western border, notably including the foothills of the Carson Range. At mid-elevations, the dry forest is composed of a mix of conifers, including California white fir, incense cedar, Jeffrey pine, with an understory of sagebrush, antelope bitterbrush, and manzanita. At elevations between 7500-9500 ft, heavy snowfall and summer droughts favours vegetation adapted to season extremes like red fir, white pine and mountain hemlock. Though a relatively small area, this extension of the Sierra Nevada fauna is significant as it is not found elsewhere in the state of Nevada.

The Arizona-Mexico Plateau (Ruhlman et al. 2010) is the ecoregion with the smallest area among the five in Nevada. It is represented by an area of the Virgin Mountains at the Nevada and Arizona border, which is the western limit of the region that extends east into New Mexico. The region has semiarid grassland and desert-scrub, piñon pine uplands, and ponderosa pine at the highest elevations.

## Materials and Methods

### Specimen sources and identification methods

Specimens examined were from the following institutions: Cornell University Insect Collection (CUIC), Ithaca, NY; Essig Museum of Entomology (EMEC), University of California, Berkeley; Monte L. Bean Life Science Museum, Arthropod Collection, Brigham Young University (BYUC); California Academy of Sciences, San Francisco, CA (CAS) including the former University of Nevada, Las Vegas (UNLV) holdings now at CAS; the teaching collection held at the Great Basin National Park (GBNP); the Oregon State University Arthropod Collection, Corvallis, OR (OSAC); and the collection of Peter W. Messer, Wisconsin (PWMC). Additional records were sought from the Symbiota Collections of Arthropods Network (SCAN) at http://symbiota4.acis.ufl.edu/scan/portal and Bug Guide at http://bugguide.net/.

We collected additional specimens in the GBNP from 10-15 June 2015 and collections were made in other regions of Nevada by K. Will in June of 2009, 2012, 2013, and May 2016. Collecting methods used included ramp traps, pitfall traps, UV light traps, leaf litter sifting, or hand collected during the day or at night using headlamps.

The primary identification references used were Lindroth’s Ground Beetles of Canada and Alaska, parts 1-6 (Lindroth 1961, Lindroth 1963, Lindroth 1966, Lindroth 1968, Lindroth 1969a, Lindroth 1969b); American Beetles, chapter 6 (Ball and Bousquet 2000) and published genus level treatment published since Lindroth’s work, as appropriate. EMEC and CAS were the primary comparative reference collections. Except as noted, taxonomic names follow Bousquet (2012). Specimens in EMEC and CAS that are identified by the revisor of a group, e.g. G. Noonan for various Harpalini or F. Heike for Zabrini, were accepted as authoritatively identified. Several authoritative records are in litteris from various sources as noted with specimen records. All other determinations were done by KWW.

## Results

We identified nearly 900 specimens from localities across the state of Nevada. This includes 79 species of carabids that represent 57 new state records, two state records previously reported online, one confirmation of a previous questionable record for the state, and report 22 records for the Great Basin National Park (GBNP) that includes three of the new state records. Nearly half, 26 of 57, species newly reported for Nevada are shared with three or more adjacent states.

### Specimen occurance data

For all new state records and GBNP records see Suppl. material 1 for the complete specimen occurance data. Full data are also published in GBIF DOI: http://doi.org/10.15468/dl.stpda0. Also under each species entry the species are linked to a query of the Essig Museum database that pulls all records for that species used in this study and allows for downloading and mapping of those records.

### Species Accounts

**Notiophilini**

*Notiophilus nitens* LeConte, 1857. New state record.

The single specimen of *N. nitens* from the Lamoille Creek Power Plant Picnic area (Elko County) represents the first report of this tribe from Nevada. This is a northwestern species from the bordering states of Oregon and Idaho. Species of notiophilines are day-active predators. *Notiophilus nitens* is known to be found in open grasslands in hilly country and is tolerant of dry conditions, and possibly xerophilus. The Nevada record represents the known southern limit of the species. Lindroth (1961) provides a key to species and ecological information.

**Loricerini**

*Loricera pilicornis pilicornis*(Fabricius, 1775). Report of online record.

A Holarctic distributed species that can be very common seasonally, near water or on wet, organic-rich mud. It is already reported from all the states surrounding Nevada and so was expected to be found. The single specimen represented as an image posted to BugGuide is all that is known to us. http://bugguide.net/node/view/898466 “South Fork Lake, Elko County, Nevada, USA, June 17, 2004” Image submitted by M. Romero, identification by P. Messer. Accessed 30 June 2016. Keys to species and ecological information were published by Ball and Erwin (1969) and Lindroth (1961).

**Carabini**

*Calosoma (Camegonia) prominens* LeConte, 1853. New state record.

This southwestern species is common in the bordering states of Arizona and California. It is notibly common at lights in Arizona. The single specimen from the Toiyabe Mountains (Nye County) is the northernmost record. These beetles are predaceous, nocturnally active, and powerful fliers. Gidaspow (1959) provides a key to species.

*Carabus (Tanaocarabus) taedatus agassii* LeConte, 1850. GBNP record.

We found beetles of this widespread, fairly common species under rocks during the day and walking at night in open, rocky coniferous forest sites from 2000-3000m. A key to species and ecological information was provided by Lindroth (1961). A discussion and reference regarding the various subspecies is provided by Bousquet (2012).

**Cicindelini**

Tiger beetles have been well treated, including taxa from Nevada, by Pearson et al. (2006). The numerous subspecies and variants, and the different genus-level concepts used in collections we studied make identification challenging. We did note what appear to be specimens of *Cicindela willistoni pseudosenilis* Horn, 1900, which would represent a new record for Nevada. However, it seems most likely that these specimens are an intergrade or variant of *Cicindela willistoni echo* Casey, 1897, which is a subspecies found in Nevada. It is likely that a focused effort might turn up new species or new records, but this was beyond the scope of our study.

**Clivinini**

*Clivina (Clivina) punctulata* LeConte, 1852. New state record.

This species is found throughout California. They are found along ponds and riparian habitats and probably nocturnal and fossorial, as is typical for the genus. The two specimens from the Humboldt River, above Rye Patch Reservoir (Pershing County) are the easternmost records. Bousquet (1997) provides a key to species.

*Schizogenius (Schizogenius) depressus* LeConte, 1852. New state record.

This species is known from all the states surrounding Nevada and so is an expected part of the fauna. They are found among the gravel at the edge of streams in fairly open habitat. Whitehead (1972) provided a key to species and ecological notes.

**Scaritini**

*Scarites (Scarites) subterraneus* Fabricius, 1775. New state record.

This is a widespread species known from the neighboring states of California and Arizona. It is a eurytopic species that is tolerant of disturbance and frequently found in agricultural settings. The 11 specimens we studied are all from in or near Las Vegas and probably its occurrence in the state is due to human transport. Bousquet and Skelley (2010) provide a key to species.

**Patrobini**

*Diplous (Platidius) aterrimus* (Dejean, 1828). New state record.

A decidedly northern species known from the neighboring states of Oregon, Idaho, and Utah that is found on the gravel shores of fast running streams and rivers. The four specimens we examined include three from the east side of the Sierra Nevada Mountains and one from the Spring Mountains. The Spring Mts. record is a significant southern range extension, but that range is known for other isolated populations such as the recently described Nebria (Catonebria) baumanni Kavanaugh, 2015. Lindroth (1961) provides key to species and ecological information.

*Diplous (Platidius) filicornis* (Casey, 1918). New state record.

Known from the adjacent states of Oregon and northern California that is found along fast, cold flowing streams. Records from Washoe Co. are from the east side of the Sierra, which is likely the eastern limit of its distribution. Key to species and ecology provided by Lindroth (1961).

*Patrobus fossifrons*(Eschscholtz, 1823). New state record.

A decidedly western beetle, previously known from California, Oregon, Idaho, and Utah. A very hygrophilus species found on lake shores and along slow moving water under debris, e.g. dead catatail (*Typha* sp). Lindroth (1961) provides key to species and ecological information.

**Trechini**

**Figure 1.**
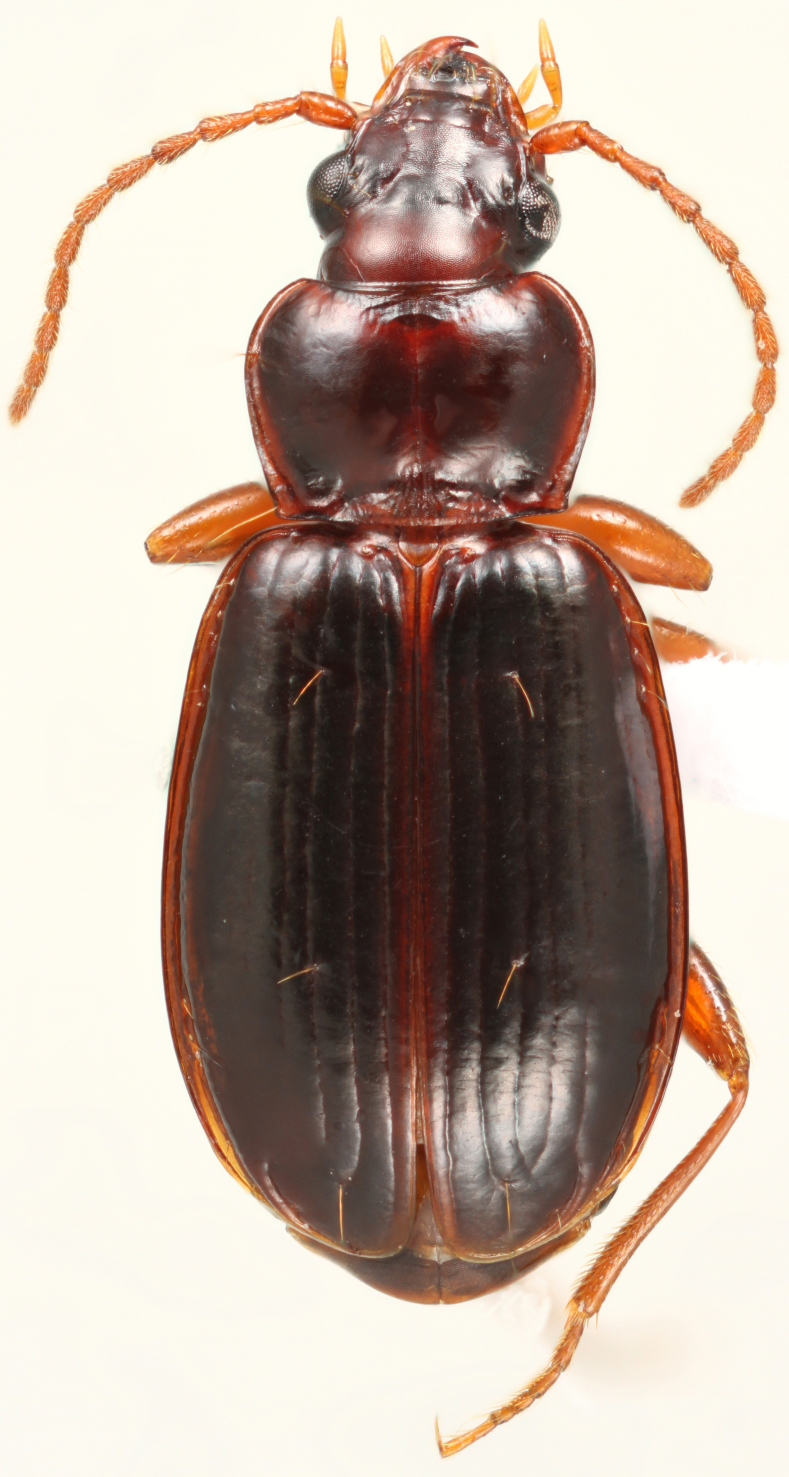
Trechus (Trechus) tenuiscapus Lindroth, 4.9 mm long male collected along the Bristelcone Pine trail, GBNP (EMEC344626). This species was found during the study and is a new state record for Nevada from Great Basin National Park.

*Trechus (Trechus) tenuiscapus* Lindroth, 1961. New state record and GBNP record. Fig. 1

A species found in the northwest with records known for the adjacent states of Oregon and Idaho. Specimens from our sampling in the GBNP were found in open conifer forest above 2300 m. Beetles were found during the day in leaf litter and active at night. This is a significant southern expansion of the known range for this species.

**Bembidiini**

Species of *Bembidion* can often be identified using the keys provided by Lindroth (1963). However, many species are difficult to identify and a considerable amount of confusion still surrounds many names proposed by T.L. Casey for western species. Confident identification of the specimens that are the basis for the records presented here was only possible with assistance and/or direct identifications provided by D.R. Maddison and J. Sproul (Oregon State University), who are presently revising the genus. Lindroth (1963) also gives ecological information for most species.

*Bembidion (Bembidion) mutatum* Gemminger & Harold, 1868. New state record.

A species with a transcontinental distribution in the north known from the neighboring states of Idaho and Utah. Recorded from Douglas County.

*Bembidion (Eupetedromus) incrematum* LeConte, 1860. New state record.

A Holarctic species, known from the adjacent states of California, Idaho, and Oregon. Specimens collected on the muddy shore of a man-made reservoir in Lander County.

*Bembidion (Furcacampa) fuchsii*Blaisdell, 1902. New state record.

A northwestern species found in the adjacent states of California, Oregon, and Idaho. Found in wet meadow with light cover of *Populus* and *Salix* in the Ruby Mountains, Elko County.

*Bembidion (Furcacampa) versicolor* (LeConte, 1847). New state record.

A widespread species that can be very abundant in a variety of wetland habitats. Previously known from the adjacent states of Idaho and Oregon.

*Bembidion (Hirmoplataphus) concolor* (Kirby, 1837). GBNP record.

A transcontinental North American species found near water. While Lindroth (1963) notes that they are found on bare gravel or coarse sand near running water or on lake shores, all specimens collected in GBNP were under rocks and debris near the water in a seasonal lake at about 3000 m elevation in rocky, clay soil.

*Bembidion (Hirmoplataphus) quadrulum* LeConte, 1861. New state record.

A western species, known from all states bordering Nevada and so is an expected species in the fauna. Collected in Elko Co., along Lamoille Creek above 2100 m elevation.

*Bembidion (Liocosmius) horni*Hayward, 1897. New state record.

Found across southern California, Arizona, and Utah.

*Bembidion (Notaphus) approximatum* (LeConte, 1852). Confirmation of state record.

Reported from the adjacent states of California and Oregon. Specimens previously attributed to this species were apparently misidentifications (Bousquet 2012, Lindroth 1963). Here we report on 33 confirmed specimens from Nye, Elko, and Lincoln Counties.

*Bembidion (Notaphus) graphicum* Casey, 1918. New state record.

A fairly widespread species previously known from the adjacent states of Arizona, Oregon, and Utah.

*Bembidion (Notaphus) intermedium*(Kirby, 1837). New state record.

A transcontinental, primarily northern species not reported from any states adjacent to Nevada. There are records from Montana, Wyoming, and Colorado. Found in sandy habitat along the Humboldt River.

*Bembidion (Notaphus) nubiculosum* Chaudoir, 1868. New state record.

Found in the southwestern US, known from the adjacent states of Arizona and California.

*Bembidion (Notaphus) obtusangulum* LeConte, 1863. New state record.

A midwest to western US species distributed generally in the north and in its southern limited extended along the Rocky and Sierra Mountains. Reported from the adjacent states of California, Oregon, Idaho, and Utah.

*Bembidion (Notaphus) umbratum* (LeConte, 1847). New state record.

In collections, specimens identified as *B. umbratum* may be *B. variolosum* (Motschulsky, 1859), which is maintained as a junior synonym by Bousquet (2012) following Lindroth (1963). These are distinct species (D.R. Maddison in litt.) with confirmed specimens of both species known from Nevada. *Bembidion umbratum* is found in the adjacent states of California, Oregon, and Idaho.

*Bembidion (Peryphodes) ephippigerum* (LeConte, 1852). New state record.

Previously only reported from California. The single specimen from Washoe Co. is a slight expansion eastward of the species as it was known from several eastern California counties in the Sierras.

*Bembidion (Peryphus) nevadense* Ulke, 1875. GBNP record.

A western North American montane species. Specimens from GBNP were collected near small and medium size flowing water and, most abundantly, under rocks and debris near the water in a seasonal lake at about 3000 m elevation in rocky, clay soil.

*Bembidion (Peryphus) striola* (LeConte, 1852). New state record.

Reported previously from California and Oregon primarily in coastal counties.

*Bembidion (Plataphus) laxatum* Casey, 1918. New state record. GBNP record.

Reported previously from California and Washington, with an unconfirmed record in British Columbia. Six specimens collected above 3000 m elevation, on wet, rocky, open soil around the shore of Teresa Lake, Great Basin National Park, White Pine County.

*Bembidion (Testediolum) nebraskense* LeConte, 1863. GBNP record.

A fairly common western North American species found in the park at around 2100m elevation along Snake Creek near a few pools of water.

*Bembidion (Testediolum) obscuripenne* Blaisdell, 1902. New state record.

A western montane species, previously known from the adjacent states of California and Oregon. Found in Lander County along a small stream in open habitat.

*Bembidion (Trepanedoris) acutifrons* LeConte, 1879. New state record.

A western species, known from the adjacent states of Oregon and Utah. The single specimen we examined was collected near the Humboldt River in Eureka Co., but its exact habitat is not known.

*Bembidion (Trepanedoris) anguliferum* (LeConte, 1852). New state record.

Previously only reported from California.

*Elaphropus (Barytachys) anthrax* (LeConte, 1852). New state record.

The species was previously reported from the adjacent states of California, Oregon, and Idaho. Hayward (1900) provides a key to species.

*Elaphropus (Barytachys) conjugens* (Notman, 1919). New state record.

This species was known only from southern Arizona and the Nye and Clark County records are a significant extension northward. Hayward (1900) provided a key to species (as *Tachys trechiformis* Hayward, 1900).

*Elaphropus (Barytachys) dolosus* (LeConte, 1848). New state record.

A widely distributed species previously known from the adjacent states of California and Arizona found on the sandy banks of rivers and lakes. Lindroth (1963) provides a key to species and ecological information.

*Polyderis rufotestacea* (Hayward, 1900). New state record.

A western species, known from almost all states adjacent to Nevada; Arizona, California, Oregon, and Idaho. Hayward (1900) provides a key to species.

**Psydrini**

*Psydrus piceus* LeConte, 1846. GBNP record.

Distributed transcontinentally in the north but restricted to the west in the southern part of its range. The single specimen collected in the GBNP was found under the bark on a log of an unidentified conifer. These beetles are always found under bark in deadwood and they are regularly collected in the Californian Sierras under the bark of dead *Pinus* and *Pseudotsuga*. The pygidial gland secretions emitted when they are disturbed are extremely pungent. Lindroth (1961) provided a key to species.

**Brachinini**

*Brachinus (Neobrachinus) elongatulus* Chaudoir, 1876. New state record.

Often found to be extremely common along the shores of lakes and streams. Known previously from the adjacent states of Arizona, California, and Oregon. Flight wing is full and they are frequently attracted to lights. Erwin (1970) provides a key to species and ecological information.

*Brachinus (Neobrachinus) phaeocerus* Chaudoir, 1868. Report of online record.

A southern species, known from as far west as the adjacent state of Arizona. This record pushes the distribution northward. The single specimen represented as an image posted to BugGuide is all that is known to us. http://bugguide.net/node/view/725859 “Las Vegas in the Red Rock Canyon National Conservation Area, Clark County, Nevada, USA April 23, 2012” image contributed by D. Lund in November 2012. Identification by T.L. Erwin. Accessed 23 June 2016. Erwin (1970) provides a key to species and ecological information.

*Brachinus (Neobrachinus) quadripennis* Dejean, 1825. New state record.

A very widespread species known from California, Oregon, Idaho, and Utah. Its occurrence in Nevada is expected. Erwin (1970) provides a key to species and ecological information.

**Chlaeniini**

*Chlaenius (Chlaeniellus) obsoletus* LeConte, 1851. New state record.

A southwestern species previously reported from California and Arizona. Sometimes found along medium sized streams. Bell (1960) provides a key to species.

*Chlaenius (Chlaeniellus) pennsylvanicus blanditus* Casey, 1920. New state record.

The nominate subspecies is transcontinental in the north and so is found in the adjacent states of Oregon and Idaho, while *C. pennsylvanicus blanditus* is from Arizona and Utah. This subspecies is more of a green color and stouter in form but otherwise is little different than the nominate. Like most *Chlaenius* species, *C. pennsylvanicus blanditus* is hygrophilus and found near water, probably slow-flowing or standing water. Key to species and subspecies is provided by Bell (1960).

**Galeritini**

*Galerita (Progaleritina) lecontei lecontei* Dejean, 1831. New state record.

The northern subspecies of a southern species, (*G. lecontei bicoloripes* Reichardt is found in central Mexico) known from the adjacent states of Arizona and California. These beetles often come to lights and are nocturnally active in various types of mesic woodlands. Lindroth (1969a) provides a key to species.

**Harpalini**

*Bradycellus (Liocellus) nitidus* (Dejean, 1829). New state record.

Known from all the states surrounding Nevada except for Idaho and so is an expected part of the Nevada fauna. Beetles are found along seasonal streams under rocks and in gravel, but nothing is known about their life history. Fall (1905) and Casey (1924) provide keys to species and descriptions, however, the subgenus Liocellus is in need of revision. Lindroth (1968) includes this species in his key as well.

*Bradycellus (Stenocellus) rivalis* LeConte, 1858. New state record.

This is a fairly common species in southern California and is also reported from Arizona. Sometimes it appears abundantly at lights in California. Its habits are unknown, but probably like other *Bradycellus* they are hygrophilus. Lindroth (1968) includes this species in his key.

*Notiobia (Anisotarsus) terminata* (Say, 1823). New state record.

A very widespread species, but only reported from Arizona among the states adjacent to Nevada. The single specimen we studied was from an urban area in the southern part of the Las Vegas region. As these beetles are known to thrive in disturbed habitats, this record may be due to human transport and it is unknown if an endemic or naturalized population occurs in Nevada. Lindroth (1968) has a key to species and ecological notes.

Selenophorus (Selenophorus) famulus Casey, 1914. New state record.

This species is known from southern California and Arizona. A record for a single female specimen from Mesquite, NV, Clark County was sent to us by P. Messer (in litt.). The specimen is deposited in his collection. No published keys cover this or many species of *Selenophorus* and a revision of the group is much needed. If this species is typical for the genus in its ecology, then it is an arid habitat species, found in sandy soils with sparse vegetation.

*Stenolophus (Agonoderus) comma* (Fabricius, 1775). New state record.

An extraordinarily widespread species, often extremely abundant and attracted to lights. It is known from every state adjacent to Nevada and so it is only surprising that it had not been recorded from Nevada previously. Lindroth (1968) provides a key to species and ecological notes.

*Stenolophus (Stenolophus) fuliginosus* Dejean, 1829. New state record.

A widespread species in the middle latitudes of North America previously reported from California, Oregon, and Idaho. Beetles are found on the shore of slow or still waters among *Carex* and *Typha*. The key by Lindroth (1968) covers this species.

*Stenolophus (Stenolophus) ochropezus* (Say, 1823). New state record.

Arguably one of the most common and widespread species of carabid beetle in North America. Reported from the adjacent states of Arizona, California, and Utah. Common on wet mud along the shoreline of still waters and in other wet areas with vegetation cover. Lindroth (1968) provides a key to species and ecological notes.

*Bradycellus (Stenocellus) congener* (LeConte, 1847). GBNP record.

A common, transcontinentally distributed species. The key by Lindroth (1968) covers this species.

*Discoderus amoenus*LeConte, 1863. GBNP record.

This species is found from Wyoming to southern California, and being xerophilic, is relatively common in Nevada. The genus is in need of revision. Casey (1914) covers many species, but the work is incomplete.

*Harpalus (Opadius) fraternus* LeConte, 1852. GBNP record.

A fairly common species found in sites across the park. Collected in pitfall traps, headlamp searching at night and under woody debris during the day. All specimens collected in GBNP and others studied (EMEC) from Nevada are from stands of aspen or aspen mixed with either willow or fir. This differs from Lindroth’s description of the ecology of the species further north, as being “[i]n dry, open country with scarce vegetation, often on sandy soil” (Lindroth 1968).

*Harpalus (Harpalobius) fuscipalpis* Sturm, 1818. GBNP record.

A Holarctic species. One specimen was collected near the Baker Creek Campground in rocky, lightly vegetated habitat at night. Key to species and ecology provided by Lindroth (1968) as *Harpalellus basilaris* Kirby.

*Harpalus (Harpalus) ellipsis* LeConte, 1847. New State record.

Known previously from adjacent states of Arizona, Utah, Idaho, and Oregon. Key to species by Lindroth (1968) and Noonan (1991). This species can only be confidently separated from *Harpalus obnixus* Casey, a species previously reported from Nevada, by examination of the male genitalia.

*Harpalus opacipennis* (Haldeman 1843). GBNP record.

A widespread North American species. Found throughout the park at 2000-2400 m elevation by pit fall traping and night searching in grassy open habitat or areas of light, low brush. Key to species provided by Lindroth (1968) who also notes this species is common on sand and gravel soils in areas of sparse vegitation.

**Platynini**

**Figure 2.**
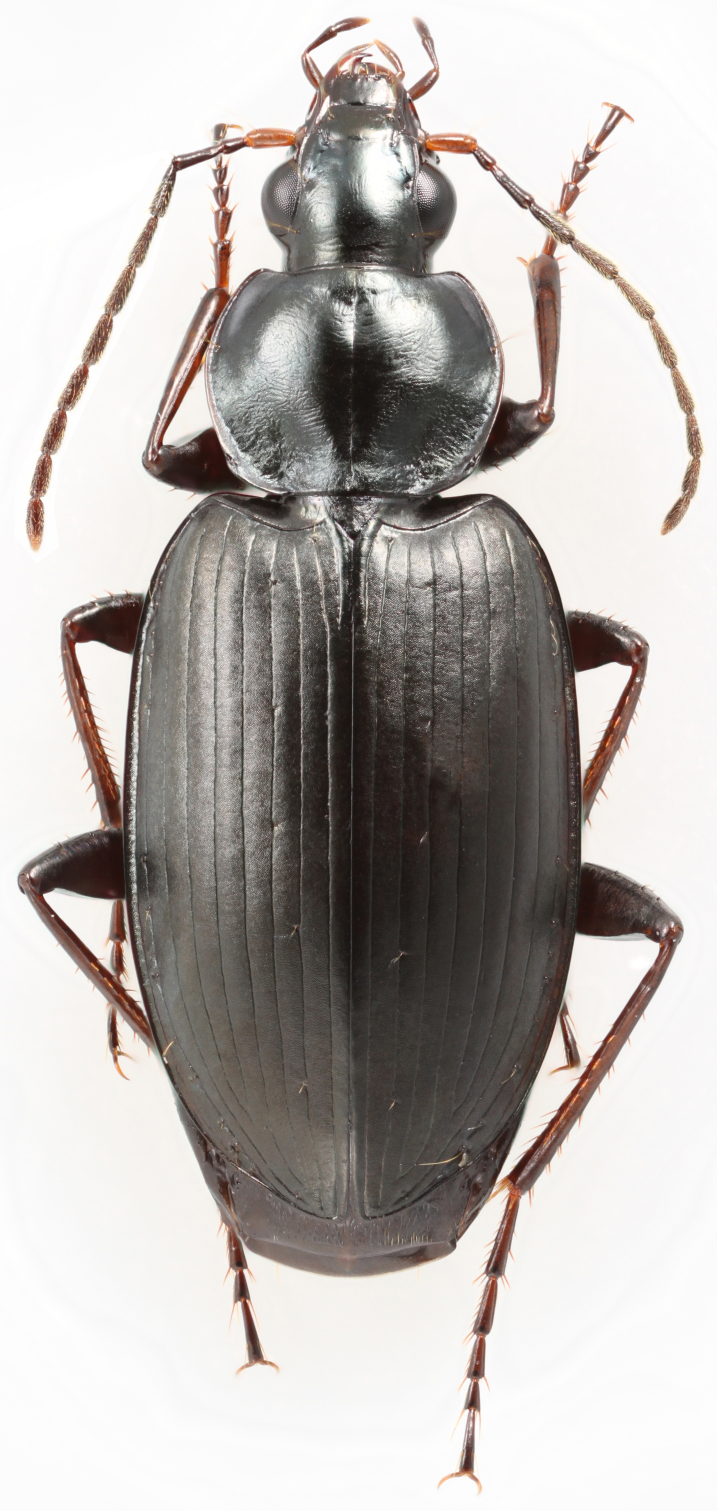
Agonum (Agonum) placidum (Say), a 10 mm long female collected in the Grey Cliff area of the GBNP (EMEC344764). This species was found during the study and is a new state record for Nevada from the Great Basin National Park.

*Agonum (Agonum) placidum* (Say, 1823). New state record and GBNP record. Fig. 2

A widespread species known from all the states surrounding Nevada and so an expected part of the fauna. We collected specimens in the GBNP while headlamp searching at night, in sites at around 2000 m elevation, along streams, looking under rocks. A specimen is also known from the park at over 3000 m elevation near Stella Lake. Other records for *A. placidum* are on the east slope of the Sierras. Liebherr (1994) provides a key to species and Lindroth (1966) includes ecological notes.

*Agonum (Europhilus) gratiosum* (Mannerheim, 1853). New state record.

This species has a northern Holarctic distribution and has been found in the neighboring states of California, Oregon, and Utah. These beetles are found in fairly open habitats with moist soils, but not necessarily close to water. However, specimens from Ruby Lake (Elko Co.) were found near the lake under dead *Typha*. Liebherr (1994) provides a key to species and Lindroth (1966), Lindroth (1969a) includes ecological notes.

*Agonum (Platynomicrus) nigriceps* LeConte, 1846. New state record.

A Holarctic, decidedly northern species only reported from Idaho of the adjacent states. This is a very hygrophilous species found among and on the plants growing in the water. Beetles can be readly collected by treading the vegetation into the water. The eight specimens we examined from the Humboldt River area in Pershing County seem a modest southward extension of this northern species. The single specimen from Las Vegas Valley, in Clark County is a remarkable southern record. Liebherr (1994) provides a key to species and includes ecological notes.

*Rhadine* sp. GBNP record.

A single specimen of *Rhadine* was collected in the Baker Creek Campground latrine. Significant effort was made to find additional specimens in the area, but none were found. The taxonomy of the genus is in need of revision. In addition to the single specimen we sampled during the study, numerous specimens were seen in collections, but they cannot be confidently identified. The genus includes both cave and surface dwelling species (Gómez et al. 2016).

**Pterostichini**

*Pterostichus (Pseudomaseus) luctuosus* (Dejean, 1828). New state record.

A widespread and relatively commonly collected species in the north and middle latitudes of North America, especially in the east. Of the states surrounding Nevada, it is only reported from Idaho. It is a decidedly hygrophilus and can be found near water and in very wet, marsh areas. The habitat where the Nevada specimens were collected, Ruby Lake (Elko Co.), is very typical for them. Lindroth (1966) provides a key to species and ecological notes.

*Pterostichus (Hypherpes) protractus* LeConte, 1860. GBNP record.

One of the most widespread species of the subgenus, ranging from northern New Mexico to Alberta, Canada, west to California. Found throughout Nevada in coniferous forests from near treeline to 2000 m (and probably lower) elevation. At lower elevations confined to riparian habitat under woody debris and in thick leaf litter. These nocturnal predatory beetles were the most common species found during our sampling at GBNP. They were significantly abundant in areas with ponderosa pine and fir. Lindroth (1966) provides a key to species, however, the key does not cover the numerous southern species in the subgenus, which is in need of revision.

*Pterostichus (Bothriopterus) lustrans* LeConte, 1851. GBNP record.

A western North American species found in numerous locations across Nevada. In the GBNP specimens were collected from multiple sites in open vegetation along the riparian corridor, though not in tight association with the stream. Lindroth (1966) provides a key to species.

**Sphodrini**

*Synuchus dubius* (LeConte, 1854). GBNP record.

A central southwest species reported from the bordering states of Arizona and Utah. Specimens examined are from three general localities in the eastern part of the state. We collected specimens from several locations in the Great Basin National Park by searching the forest leaf litter at night in areas above 2000 m. These are flightless beetles and other localities for this species are at similar elevations, suggesting significantly disjunct populations. Lindroth (1966) provides a key to species.

*Laemostenus (Laemostenus) complanatus* (Dejean, 1828). New state record.

A globally subcosmopolitan species that is an adventive and synanthropic. Known from the adjacent states of California and Oregon, especially in near-coastal regions in urban areas such as watered lawns and parks. The single specimen we studied is only labeled Clark Co., but if there is an established population it would probably be in human maintained habitat in the Las Vegas area. Lindroth (1966) provides a key to species.

*Calathus (Acalathus) advena* (LeConte, 1846). GBNP record.

A widespread forest, leaf litter species that occurs in the northeastern part of Nevada. In the conifer forest habitat of the GBNP we found this to be the most commonly encountered species. Frequently found with *Pterostichus protractus*. Lindroth (1966) and Ball and Nègre (1972) provide keys to species and ecological notes.

**Zabrini**

*Amara (Amarocelia) farcta* LeConte, 1855. GBNP record

A western North American species found in various open habitats near water. All specimens collected in GBNP were under rocks and debris near the water in a seasonal lake at about 3000 m elevation. Lindroth (1968) provides a key that includes this species.

*Amara (Bradytus) lindrothi* Hieke, 1990. GBNP record.

Known from localities widely distributed across western North America. GBNP is near its southern limit. Specimens were collected in open conifer forest between 3000 and 3200 m elevation, on wet ground, under rocks and debris. Identification is possible using the description and illustrations provided by Hieke (1990).

*Amara (Celia) idahoana* (Casey, 1924). New state record.

Found in the west, in states north of Nevada, including the adjacent states of Idaho and Oregon. Records from Nevada extend the range significantly southward. However, while the record from near Weeks (Lyon Co.) is in an irrigated area near the Carson River, the record from Nye County is in a desert habitat and may be a mislabeled specimen. Lindroth (1968) provides a key to species and ecological notes.

*Amara (Celia) sinuosa* (Casey, 1918). New state record.

A northern species, known from the adjacent states of Idaho and Utah. The single specimen we examined was collected in 1936 and labeled Governors Canyon, Nevada. We have been unable to find a match for that exact locality. Lindroth (1968) provides a key to species and notes that the species is found “[o]n open, dry, sandy or gravelly ground.”

*Amara (Curtonotus) carinata* (LeConte, 1847). New state record.

A widespread species found in all states adjacent to Nevada and so an expected part of the carabid fauna. Beetles are found in grasslands, frequently near alkaline habitats. Lindroth (1968) provides provides a key to species and ecological notes.

*Amara (Paracelia) quenseli quenseli* (Schönherr, 1806). GBNP record.

A Holarctic species that is frequently common. Specimens were collected across the GBNP, during the day and at night in open, grassy habitat. Lindroth (1968) provides a key that includes this species.

**Lebiini**

*Apristus pugetanus* Casey, 1920. New state record.

A western North American species, already known from all the states surrounding Nevada and so it is an expected member of the fauna. The beetles are found in gravel and sand along rivers, creek and springs, although they are often found some distance from the water. They are frequently day active. Lindroth (1968) provides a key to species.

*Axinopalpus biplagiatus* (Dejean, 1825). New state record.

A widespread species found in all states adjacent to Nevada and so an expected part of the carabid fauna. Little is known about the ecology and habits of this species but it has been collected in a variety of open habitats and of the ones we studied from Nevada, one was taken from Joshua Trees. Lindroth (1968) discusses this species, but there are more species in the region and there is no revision of the group. Comparison with types and original descriptions must be used for identification.

*Axinopalpus fusciceps* LeConte, 1851. New state record.

A western species, found in the middle latitudes of North America, south into Guatemala. Know from scattered records in the adjacent states of Arizona, California, and Idaho. Nothing is known of this species habits and there is no key to species for the genus. For identification recourse to original descriptions is needed.

*Cymindis (Tarulus) arizonensis* Schaeffer, 1910. New state record.

This species is known from southwestern California and Arizona. The record from Clark Co. is a slight expansion of its range. Lindroth (1968) provides a key to species and discussion of the taxonomic difficulties related to this species.

*Lebia (Lebia) perita* Casey, 1920. New state record.

A western species, known previously from the adjacent states of California, Oregon, and Idaho. One specimen from Spencer Hot Spring (Landers County) was taken on whiteflower rabbitbrush (*Chrysothamnus albidus*). This marks the easternmost record for the species. Madge (1967) provides a key to species and distributional data.

**Zuphiini**

*Pseudaptinus (Thalpius) rufulus* (LeConte, 1851). New state record.

A western North American species, known previously from Arizona, California, and Oregon. Little is known about the habits of this species. A key to species of the subgenus Thalpius was published by Messer (2011).

**Pseudomorphini**

*Pseudomorpha castanea* Casey, 1909. GBNP record.

This species was previosuly reported from California, Oregon, and Utah. As far as known, pseudomorphines are myrmecophiles and ovoviviparous (Liebherr and Kavanaugh 1985). Notman (1925) includes a key to species, however, many undescribed species exist (T.L. Erwin, in litt.) and the genus is in need of revision.

## Discussion

**Figure 3.**
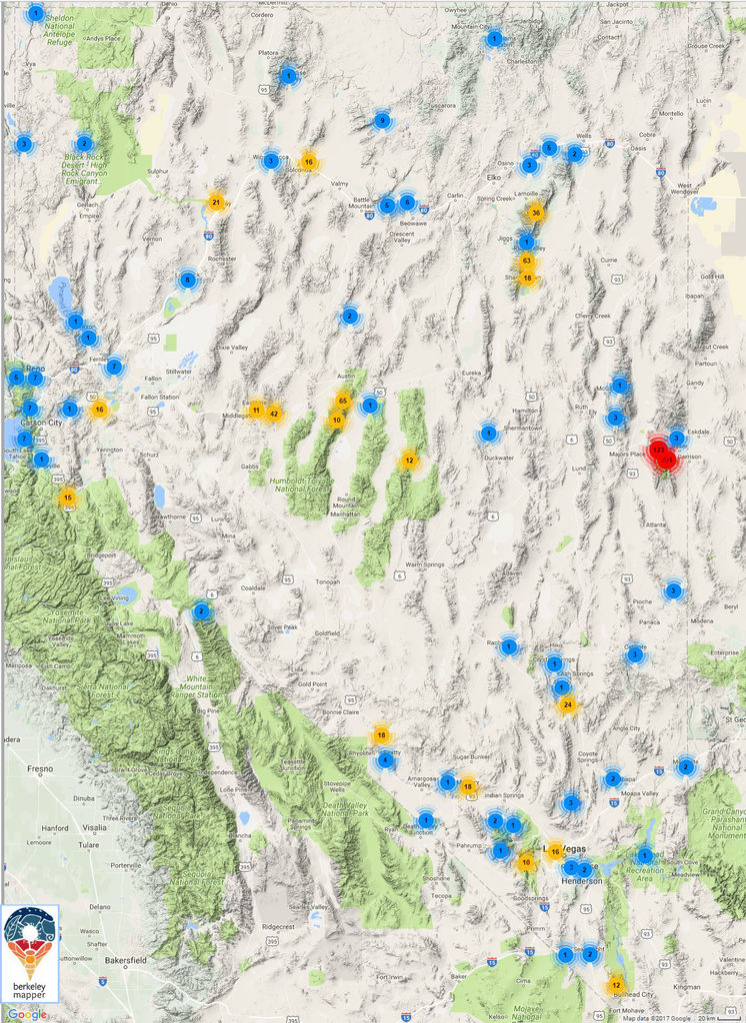
Clustered distribution of specimens of carabid species reported here. Plot generated by Berkeley Mapper ver. 2.0 http://berkeleymapper.berkeley.edu/ Complete specimen records are included in Suppl. material 1.

We identified nearly 900 specimens from localities across the state of Nevada (Fig. 3). Nearly half, 26 of 57, species newly reported for Nevada are shared with three or more adjacent states. Only Bembidion (Notaphus) intermedium is a trans-state extralimital for Nevada. There is no strong pattern in the regional similarity of the new records, i.e., about half of the species have relatively northern distributions that include Oregon, Idaho and northern California and remaining species are either more southern or widespread. Qualitatively, the current list of species of carabids found in Nevada appears to reflect the habitats that are available, with many species that are known to be riparian and seasonal wetland associates or obligate open habitat grassland species.

The only non-native species found during our study was Laemostenus (Laemostenus) complanatus and it is not clear that this species has established. It is strongly associated with human development in other areas where it has been introduced (e.g., California) and has not shown a tendency to move into undisturbed habitats.

The 57 species we report here grows the list of carabid species for Nevada to 299, much closer to its ecologically comparable neighbors Idaho (338 species) and Utah (323 species) (numbers from Bousquet (2012)). It is expected that Oregon (478 species), with its exceptionally diverse coastal fauna and California (646 species), with its much larger area and multiple bioregions, will have many more species than Nevada. In fact, it is likely that all of these western states have many dozens, perhaps hundreds of species yet to be reported. For California, estimates of the true fauna is over 800 species (K. Will and D.H. Kavanaugh, unpubl.).

## Conclusions

While ongoing monographic research and targeted collecting efforts for surveys, bioblitzes, and ecological monitoring (Kao et al. 2012) are critical to collecting baseline data on faunal distributions, our museum and university collections hold many decades of data on carabids that have yet to be exposed. Our study drew on previously collected material spanning more than 100 years (1913- present). Recent efforts to digitize insect and spider collections (Baker 2011, Hill et al. 2012, iDigBio 2017) and online communities for sharing images (e.g. BugGuide 2017) are greatly accelerating the process of discovery and exposure of more complete species distributions. We anticipate a surge in the number of species known for Nevada and other states in western USA in the near future because of this.

## Supplementary Material

Supplementary material 1 Occurence data for new state records and GBNP records for carabids of Nevada Data type: occurences
Brief description: All specimen records recorded in the EssigDB for species that are new state records and GBNP records for carabids of Nevada
File: oo_121705.csv Will, Madan, Hsu
